# Rice protein improves adiposity, body weight and reduces lipids level in rats through modification of triglyceride metabolism

**DOI:** 10.1186/1476-511X-11-24

**Published:** 2012-02-13

**Authors:** Lin Yang, Jia-Hou Chen, Jie Lv, Qiong Wu, Tong Xu, Hua Zhang, Qiao-Hong Liu, Hong-Kun Yang

**Affiliations:** 1 Department of Food Science, School of Food Science and Engineering, Harbin Institute of Technology, 73 Huanghe Road, Harbin 150090, China; 2 Heilongjiang Provincial Environmental Monitoring Central Station, Harbin 150056, China; 3 School of Life Science and Biotechnology, State Key Laboratory of Urban Water Resource and Environment, Harbin Institute of Technology, Harbin 150001, China; 4 Heilongjiang Provincial Hospital, Harbin 150036, China

**Keywords:** Rice protein, Triglyceride, Lipogenesis, Lipolysis, Digestibility, Rats

## Abstract

**Background:**

To elucidate whether rice protein can possess a vital function in improving lipids level and adiposity, the effects of rice proteins extracted by alkaline (RP-A) and α-amylase (RP-E) on triglyceride metabolism were investigated in 7-week-old male Wistar rats fed cholesterol-enriched diets for 2 weeks, as compared with casein (CAS).

**Results:**

Compared with CAS, plasma concentrations of glucose and lipids were significantly reduced by RP-feeding (*P *< 0.05), as well as hepatic accumulation of lipids (*P *< 0.05). RP-A and RP-E significantly depressed the hepatic activities of fatty acid synthase (FAS), glucose 6-phosphate dehydrogenase (G6PD) and malate dehydrogenase (MDH) (*P *< 0.05), whereas the activities of lipoprotein lipase (PL) and hepatic lipase (HL) were significantly stimulated (*P *< 0.05), as compared to CAS. Neither lipids level nor activities of enzymes were different between RP-A and RP-E (*P *> 0.05). There was a significant positive correlation between protein digestibility and deposit fat (r = 0.8567, *P *< 0.05), as well as the plasma TG concentration (r = 0.8627, *P *< 0.05).

**Conclusions:**

The present study demonstrates that rice protein can modify triglyceride metabolism, leading to an improvement of body weight and adiposity. Results suggest that the triglyceride-lowering action as well as the potential of anti-adiposity induced by rice protein is attributed to upregulation of lipolysis and downregulation of lipogenesis, and the lower digestibility of rice protein may be the main modulator responsible for the lipid-lowering action.

## Background

Obesity is an indicator for the disorder of lipid metabolism and has become a worldwide epidemic [1,2]. To prevent the occurrence of the life style-related diseases, increasing evidences suggest that dietary components can improve lipid metabolism to control body weight and reduce deposit fat [3-5]. Compared with animal protein such as casein, soy protein has exhibited a beneficial effect on lipid metabolism to improve body weight and adiposity through suppressing hepatic lipogenic enzyme activity [6-8]. However, up to now, there is not yet a comprehensive understanding for a link of anti-adiposity and the consumption of rice protein, which is another major plant protein in the world.

Rice is a staple cereal and widely consumed in the world. There is growing emphasis on the improvement of the physiological function of rice [9-12], in which the association of rice protein consumption with modulation of body weight gain and plasma cholesterol level has been extensively demonstrated in some studies [13-15]. But the precise mechanism by which rice protein affect lipid metabolism is not fully established, and the evidence on whether rice protein can regulate lipogenesis and lipolysis is lacking.

The liver plays a major role in maintaining triglyceride (TG) homeostasis through the regulation of lipogenesis and lipolysis. In the lipogenic pathway, fatty acids synthesized by the liver are concerted to triglyceride, which is packaged into very-low-density lipoprotein (VLDL) and transported into circulation [16]. In contrast, lipolysis represents the major route for the breakdown of lipids and the hydrolysis of triglyceride into free fatty acids to serve a fuel in the body, in which lipoprotein lipase (LPL) and hepatic lipase (HL) are the two major lipolytic enzymes responsible for catalyzing the hydrolysis of triglyceride component present in the circulating VLDL [17,18]. Generally, LPL is located in endothelium and HL is located in the liver. Thus, the lipogenesis and lipolysis might represent a possible mechanism for the deposition and removal of lipids throughout the circulation.

Yang *et al*. have demonstrated that lipid-lowering effects of rice proteins are mainly attributed to the inhibition of hepatic secretion of triglyceride and cholesterol via VLDLs, showing the decreased secretion of VLDL-TG is accompanied by the depressed export of VLDL-C into circulation, thereby resulting in a dramatic reduction in plasma lipids [19]. Furthermore, the regulatory effects of rice protein on fatty acid and cholesterol synthesis, which might derive from a common pool, is suggested to be co-operation because the cholesterol-lowering effect always accompanied with a triglyceride-lowering effect on the hepatic level in growing and adult rats fed by rice proteins [13]. This view is supported by some findings that both hepatic fatty acid and cholesterol synthesis are coordinately controlled by 3-hydroxy-3-methyl-glutaryl-CoA reductase (HMG-CoA reductase) and the sterol regulatory element binding protein (SREBP) family [20,21]. Thus, in light of these facts, we hypothesized that rice protein might exert an effect on triglyceride metabolism involving with the adiposity, accompanying with a hypocholesterolemic action.

To test our hypothesis, the present study was conducted to focus on the regulatory effects of rice proteins on lipogenesis and lipolysis. The key questions addressed are: (1) whether can rice protein improve fat accumulation in adipose tissue and (2) how does rice protein possess a vital function in improving triglyceride metabolism in growing rats fed cholesterol-enriched diets? In addition, extraction method for rice protein isolation is suggested to influence the lipid metabolism through the modification of protein digestibility and amino acids composition [15]. Thus, two extraction methods for preparation of rice protein, namely, an alkaline treatment and a heat-stable α-amylase degradation, were developed to evaluate and compare the physiological functions of rice proteins in this study.

## Methods

### Protein sources

Casein (CAS) (Gansu Hualing Industrial Group, Gansu, China) and rice proteins extracted from *Oryza sativa *L. cv. *Longjing *26 (Rice Research Institute of Heilongjiang Academy of Agricultural Sciences, Jimusi, China) were used as the dietary protein sources. Two methods were conducted for preparation of rice proteins, a classical extraction method with alkaline followed by precipitation with acidic solution (RP-A) [15], and a method for rice protein isolation by starch degradation using a heat-stable α-amylase (Sigma, St. Louis, Mo., USA, RP-E) [15].

### Animals and diets

The present experiments followed the same as the previous studies [15]. Animal studies used in the present work have been performed in conformity with the Guidelines of the Committee for Animal Experimentation of Harbin Medical University (SCXK20020002). 7-week-old male Wistar rats were purchased from Animal Center of Harbin Medical University (Harbin, China) and individually housed in metabolic cages in a room maintained at 22 ± 2°C under a 12 h light-dark cycle (07:00-19:00 for light). Rats were allowed free access to commercial pellets (Animal Center of Harbin Medical University, Harbin, China) for 3 days. After acclimatization, rats were randomly divided into three groups of similar body weight. Each group consisted of six rats.

All animals were fed *ad libitum *with experimental diets according to the formula recommended by American Institute of Nutrition [22]. For 2 weeks, growing rats were fed with experimental diets of 20% (as crude protein) of the respective dietary protein (i.e., RP-A, RP-E and CAS) to which 1% cholesterol and 0.25% sodium cholate were added. Diets were completed to 100% with starch. The composition of experimental diets is shown in Table 1.

**Table 1:** Composition of the experimental diets^a^

	CAS	RP-A	RP-E
		g/kg	
CAS*^b^*	229.1	-	-
RP-A*^c^*	-	231.5	-
RP-E*^d^*	-	-	251.3
sucrose	100.0	100.0	100.0
cellulose	50.0	50.0	50.0
soybean oil	70.0	70.0	70.0
β-cornstarch	487.9	488.5	468.7
mineral mix*^e^*	35.0	35.0	35.0
vitamin mix *^f^*	10.0	10.0	10.0
choline bitartrate	2.5	2.5	2.5
tert-butylhydroquinone	0.014	0.014	0.014
L-cystine	3.0	-	-
cholesterol	10.0	10.0	10.0
sodium cholate	2.5	2.5	2.5

**^a ^**AIN-93. Abbreviations used: CAS, casein; RP-A, rice protein extracted by alkaline treatment; RP-E, rice protein extracted by α-amylase

^b ^Protein content 873 g/kg, obtained from Huanling Industrial Group (Gansu, China)

^c ^Protein content 864 g/kg, prepared by our laboratory

^d ^Protein content 796 g/kg, prepared by our laboratory

^e ^Mineral mixture, AIN-93 G-MX (Nosan Corp., Yokohama, Japan)

^f ^Vitamin mixture, AIN-93-VX (Nosan Corp., Yokohama, Japan)

### Samples collection

During the feeding period, food consumption and body weight were recorded daily in the morning before replenishing the diet. Feces were collected for the final 3 d of the experimental period and air-dried at 60°C for 12 h to a constant weight and ground to a fine powder for fecal steroids determination according to Yang *et al*. [15].

At the end of the feeding period, the rats were deprived for 18 h and then sacrificed. Blood was withdrawn from abdominal vein into a heparinized syringe under anesthesia with sodium pentobarbital (50 mg/kg body weight), immediately cooled on ice and separated by centrifugation at 12,000 × g for 5 min. The plasma obtained was frozen at -20°C until analysis. After blood collection, the liver, the spleen and the kidney were excised immediately, rinsed in saline and weighed after blotted on a filter paper. The whole liver was cut into three portions and quickly freeze-clamped in liquid nitrogen and stored at -80°C until analysis.

### Plasma amino acid analysis

After deproteinization, plasma amino acids were measured using a Biochrom 30 amino acid analyzer (Biochrom, Holliston, MA, USA) according to Yang *et al*. [15].

### Analyses of plasma glucose, albumin, lipids and lipoprotein profiles

Plasma concentrations of glucose, albumin, free fatty acids (FFA), triglyceride (TG), total cholesterol (TC), low-density lipoprotein cholesterol (LDL-C) and high-density lipoprotein cholesterol (HDL-C) were measured using the commercial kits (Nanjing Jiancheng Bioengineering Institute, Nanjing, China). Plasma total protein contents were estimated according to the method of Lowry *et al*. [23]. Plasma very-low-density lipoprotein cholesterol (VLDL-C) was calculated as: VLDL-C = TC-HDL-C-LDL-C. The intra-assay coefficients of variability were less than 5%.

### Measurement of deposit fat

After blood collection, epididymal, perirenal and retroperitoneal fat were excised immediately, rinsed in saline and weighed after blotted on a filter paper. The total deposit fat was expressed as the relative weight of epididymal, perirenal and retroperitoneal fat to the total weight of rats: Total deposit fat (g/100 g wt) = (epididymal fat + perirenal fat + retroperitoneal fat) (g) × 100/final body weight (g).

### Liver lipid analysis

The lipids in the liver were extracted and purified according to the method of Folch *et al*. [24], and were analyzed as described by Yang *et al*. [15]. Briefly, 0.5 g of liver was extracted with 20 mL of chloroform/methanol (2:1, v/v). After extraction, total cholesterol, triglyceride and free fatty acids were measured with a commercial kit (Nanjing Jiancheng Bioengineering Institute, Nanjing, China). Total lipids were determined gravimetrically.

### Analyses of enzyme activities

The activities of plasma alanine transaminase (ALT), aspartate transaminase (AST), liver fatty acid synthase (FAS), glucose 6-phosphate dehydrogenase (G6PD), malate dehydrogenase (MDH), lipoprotein lipase (LPL) and hepatic lipase (HL) were determined using the methods described in the kits from Nanjing Jiancheng Bioengineering Institute (Nanjing, China). The activity of total lipase (TL) was calculated as: TL(nmol/min per mg protein) = LPL+HL.

### Determination of fecal excretion

The total fat in the feces was extracted with chloroform/methanol (2:1, v/v) and determined gravimetrically according to the method of Folch *et al*. [24]. After lipid extraction, fecal triglyceride in the extracted lipid was assayed with a commercial kit (Nanjing Jiancheng Bioengineering Institute, Nanjing, China). Fecal nitrogen content was determined by Kjeldahl method [15]. Apparent digestibility of protein was calculated as: Apparent protein digestibility (%) = (Protein intake-Fecal protein) × 100/Protein intake.

### Statistical analysis

Data are presented as means ± SEM (standard error of mean). Differences between groups were examined for statistical significance using the one-way analysis of variance (ANOVA), and then determined with the least significant difference test. The criterion for significance was *P *< 0.05.

## Results

### Body weight, food intake and organ weight

Body weight gain of growing rats were significantly reduced by 17.39% in RP-A and by 19.37% in RP-E, respectively, as compared with CAS (*P *< 0.05). No significant difference of gains in body weight was found in RP-A and RP-E (*P *> 0.05). Food intake was not significantly different among groups, suggesting that dietary protein did not affect food intake (Table 2).

**Table 2:** Body weight, food intake and organ weight of rats fed experimental diets^*a*^

	CAS	RP-A	RP-E
Initial body weight (g)	193.32 ± 3.34	192.67 ± 4.79	193.50 ± 3.95
Final body weight (g)	264.17 ± 3.77^A^	251.17 ± 3.08^B^	250.67 ± 6.66^B^
Body weight gain (g/day)	5.06 ± 0.12^A^	4.18 ± 0.21^B^	4.08 ± 0.27^B^
Food intake (g/day)	21.99 ± 0.45	20.82 ± 0.39	20.03 ± 0.22
Organ wt (g/100 g wt)			
Liver	4.04 ± 0.04^A^	3.58 ± 0.10^B^	3.49 ± 0.07^B^
Spleen	0.20 ± 0.01	0.20 ± 0.00	0.19 ± 0.01
Kidney	0.78 ± 0.03	0.78 ± 0.01	0.77 ± 0.02

*^a^*Values are means ± SEM for six rats. Values with different letters are significant different (*P *< 0.05)

After 2 weeks feeding, the visceral organs, spleen and kidney, showed the similar weights among the experimental groups (*P *> 0.05). However, the liver weights of rats fed RP-A and RP-E were significantly lower than those fed CAS (*P *< 0.05) (Table 2). Clearly, results suggest that the target of these dietary proteins is the liver rather than spleen or kidney.

### Plasma amino acids

Amino acids compositions of plasma were shown in Table 3. At the end of the feeding experiment, the plasma compositions of essential amino acids (EAAs) of rats were lower in RP groups than those of CAS (*P *< 0.05). Compared with CAS, the branched chain amino acids (BCAAs) level in the plasma was significantly decreased by 16.12% in RP-A and by 18.90% in RP-E, respectively (*P *< 0.05). Similarly, leucine was also significantly reduced by 17.06% in RP-A and by 16.82% in RP-E, respectively, as compared to CAS (*P *< 0.05). In contrast, higher contents of glycine were found in RP groups, as compared with CAS (*P *< 0.05).

**Table 3:** Plasma amino acid composition of rats fed experimental diets^*a*^

	CAS	RP-A	RP-E
		nmol/mL	
Asp	17.4 ± 0.9^A^	13.1 ± 0.3^B^	16.8 ± 1.6^A^
Thr	315.6 ± 3.3^A^	259.0 ± 13.0^B^	240.1 ± 9.0^B^
Ser	285.5 ± 7.7	292.0 ± 10.8	312.9 ± 20.1
Glu	137.4 ± 11.8^B^	143.4 ± 10.7^B^	192.5 ± 16.0^A^
Gly	396.1 ± 15.9^B^	457.4 ± 3.8^A^	494.6 ± 26.9^A^
Ala	431.6 ± 25.4	396.1 ± 19.3	425.5 ± 26.7
Val	243.7 ± 9.1^A^	206.4 ± 10.8^B^	192.5 ± 6.3^B^
Ile	129.9 ± 6.5^A^	108.9 ± 8.1^AB^	106.3 ± 8.5^B^
Leu	201.6 ± 13.1^A^	167.2 ± 9.4^B^	167.7 ± 7.0^B^
Cys	53.8 ± 2.7	49.9 ± 1.9	53.5 ± 3.3
Met	51.5 ± 2.7	45.3 ± 2.6	46.1 ± 4.7
Tyr	82.7 ± 4.9^A^	70.2 ± 5.8^AB^	63.0 ± 6.3^B^
Phe	75.4 ± 3.6^A^	69.6 ± 2.7^AB^	62.8 ± 1.7^B^
His	70.1 ± 3.6^A^	61.3 ± 1.7^B^	62.2 ± 1.3^B^
Lys	430.6 ± 30.8	444.3 ± 19.1	414.2 ± 17.1
Arg	124.0 ± 10.1	116.9 ± 2.3	105.3 ± 3.3
Pro	148.8 ± 6.3	149.3 ± 8.3	151.5 ± 5.4
Trp	71.8 ± 4.3	62.7 ± 5.6	66.7 ± 6.1

*^a^*Values are means ± SEM for six rats. Values with different letters are significant different (*P *< 0.05)

### Plasma glucose, albumin, lipids and lipoprotein profiles

As shown in Table 4, RP-A and RP-E significantly reduced plasma glucose level (RP-A: -9.99%; RP-E: -15.81%), as compared to CAS (*P *< 0.05).

**Table 4:** Plasma components of rats fed experimental diets^*a*^

	CAS	RP-A	RP-E
Cholesterol (mmol/L)			
TC	1.66 ± 0.07^A^	1.49 ± 0.05^B^	1.43 ± 0.04^B^
VLDL-C	0.25 ± 0.05^A^	0.19 ± 0.04^B^	0.16 ± 0.04^B^
LDL-C	0.35 ± 0.07^A^	0.28 ± 0.03^B^	0.25 ± 0.04^B^
HDL-C	1.07 ± 0.06	1.02 ± 0.03	1.02 ± 0.02
VLDL/HDL (mol/mol)	0.24 ± 0.06^A^	0.19 ± 0.04^B^	0.16 ± 0.04^B^
Triglyceride (mmol/L)	0.61 ± 0.05^A^	0.53 ± 0.03^AB^	0.51 ± 0.04^B^
TG/HDL-C (mol/mol)	0.57 ± 0.04	0.52 ± 0.04	0.50 ± 0.05
Free fatty acids (mmol/L)	1.12 ± 0.08^A^	0.98 ± 0.03^AB^	0.93 ± 0.03^B^
Glucose (mmol/L)	8.41 ± 0.29^A^	7.57 ± 0.25^B^	7.08 ± 0.15^B^
Total protein (g/L)	61.78 ± 2.40	59.13 ± 1.08	58.03 ± 0.62
Albumin (g/L)	30.17 ± 1.69	29.00 ± 1.26	28.93 ± 1.35

*^a^*Values are means ± SEM for six rats. Values with different letters are significant different (*P *< 0.05)

Compared with CAS, plasma TG concentration was decreased by RP feeding in this study (Table 4). However, a marked reduction in plasma TG concentration was only produced by RP-E (-16.39%) as compared to CAS (*P *< 0.05), whereas TG-lowering actions were decreased to -13.11% by RP-A in growing rats, showing no significant difference from CAS (*P *> 0.05). As for the ratio of TG to HDL, the decrease was found in RP-A and RP-E as compared to CAS (RP-A: -8.77%; RP-E: -12.28%), but there was no significant difference of TG/HDL ratio among experimental groups (*P *> 0.05).

Similar to TG concentration, plasma FFA level were also lowered by RP-A (-12.50%) and by RP-E (-16.96%), as compared to CAS. RP-E exhibited a significant reduction in FFA level (*P *< 0.05), whereas RP-A did not. To support this result, as for the major carrier of FFA from liver to blood, the concentration of albumin in plasma was decreased by 3.88% in RP-A and by 4.11% in RP-E, respectively, as compared to CAS (*P *> 0.05). Similarly, the lower level of plasma total protein was also observed in RP-A or RP-E, as compared with CAS (*P *> 0.05).

Accompanying the decreased level of TG, plasma TC concentrations were significantly lowered in growing rats fed RP-A and RP-E, as compared with CAS (*P *< 0.05), while the hypocholesterolemic effect of RP-E (TC: -13.86%) was similar to that of RP-A (TC: -10.24%).

With the decreased plasma TC, plasma VLDL-C and LDL-C concentrations were also distinctly lower in growing rats fed RP-E and RP-A than those fed CAS (*P *< 0.05), whereas HDL-C level was not significantly different among experimental groups (*P *> 0.05). As a result, the ratio of VLDL-C/HDL-C was significantly lowered by 20.83% in RP-A and by 33.33% in RP-E, respectively, compared with CAS (*P *< 0.05). Results indicated that RP-feeding caused an effective effect in alteration of cholesterol distribution, which was reflected mainly by the less concentration of LDL and VLDL in RP groups. In agreement with the previous findings [13,15], the factor lowering the cholesterol concentration in rats fed RP was the lowering of the non-HDL-cholesterol level and the ratio of non-HDL to HDL, which was a significant risk factor for obesity [25].

### Activities of plasma ALT and AST

**Figure 1:**
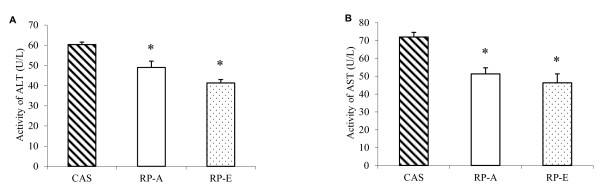
**Effects of dietary proteins on plasma activities of alanine transaminase and aspartate transaminase**. Seven-week-old male Wistar rats were fed 20% dietary proteins with the supplemental cholesterol (10 g/kg diet) for 2 weeks. (A) Alanine transaminase. (B) Aspartate transaminase. Values are means ± SEM (n = 6). Bars for each value with * were significantly different from CAS (*P *< 0.05). ALT, alanine transaminase; AST, aspartate transaminase; CAS, casein; RP-A, rice protein extracted by alkaline treatment; RP-E, rice protein extracted by α-amylase.

After 2 weeks feeding, results indicated that the activities of plasma ALT (Figure 1A) and AST (Figure 1B) were affected by the dietary proteins. The activities of ALT and AST were decreased significantly when rats were fed on RP-A (ALT: -18.78%; AST: -28.71%) and RP-E (ALT: -31.49%; AST: -35.65%), as compared with CAS (*P *< 0.05). No significant difference was found between RP-A and RP-E (*P *> 0.05).

### Hepatic lipids and deposit fat

Hepatic accumulations of total lipids, total cholesterol and triglyceride were significantly lower in rats fed RP than those fed CAS (*P *< 0.05) (Table 5), in accordance with the previous studies [13,15].

**Table 5:** Hepatic lipids and deposit fat of rats fed experimental diets^*a*^

	CAS	RP-A	RP-E
Hepatic lipids			
total lipids (mg/g liver)	206.58 ± 9.33^A^	188.53 ± 8.31^B^	170.41 ± 5.11^B^
cholesterol (μmol/g liver)	122.32 ± 6.40^A^	107.82 ± 10.33^B^	91.42 ± 6.06^B^
triglyceride (μmol/g liver)	93.36 ± 8.84^A^	77.42 ± 5.97^B^	68.68 ± 4.71^B^
free fatty acids (μmol/g liver)	53.31 ± 5.53^A^	46.52 ± 5.34^AB^	44.75 ± 2.94^B^
Deposit fat (g/100 g wt)			
total	5.44 ± 0.12^A^	4.80 ± 0.10^AB^	4.60 ± 0.15^B^
perirenal	1.85 ± 0.11^A^	1.59 ± 0.08^AB^	1.52 ± 0.06^B^
epididymal	1.67 ± 0.06^A^	1.50 ± 0.02^AB^	1.44 ± 0.03^B^
retroperitoneal	1.92 ± 0.07^A^	1.71 ± 0.08^AB^	1.64 ± 0.10^B^

*^a^*Values are means ± SEM for six rats. Values with different letters are significant different (*P *< 0.05)

The hepatic triglyceride-lowering actions induced by RP-feeding were mainly reflected by the diminished concentrations of free fatty acids. RP-A and RP-E reduced hepatic free fatty acids levels by 12.74% and by 16.06%, respectively. However, compared with CAS, a significant reduction in free fatty acids concentration was only caused by RP-E (*P *< 0.05), whereas RP-A did not (*P *> 0.05).

With the similar tendency of hepatic lipid accumulation, the deposit of perirenal, epididymal and retroperitoneal fat were inhibited by RP-A (perirenal fat: -14.05%; epididymal fat: -10.18%; retroperitoneal fat: -10.94%) and RP-E (perirenal fat: -17.84%; epididymal fat: -13.77%; retroperitoneal fat: -14.58%) (Table 5). Compared with CAS, RP-E produced a significant depress in adiposity (*P *< 0.05), whereas RP-A did not (*P *> 0.05).

### Activities of hepatic enzymes involved in lipogenesis and lipolysis

**Figure 2:**
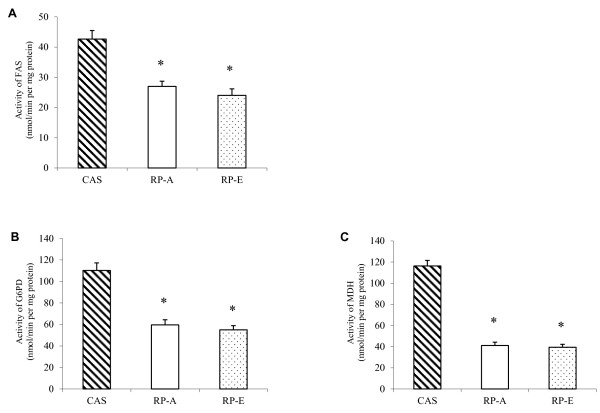
**Effects of dietary proteins on hepatic activities of lipogenesis enzymes**. Hepatic activities of fatty acid synthase (A), glucose 6-phosphate dehydrogenase (B) and malate dehydrogenase (C) in 7-week-old male Wistar rats fed cholesterol-enriched diets for 2 weeks. Values are means ± SEM (n = 6). Bars for each value with * were significantly different from CAS (*P *< 0.05). CAS, casein; FAS, fatty acid synthase; G6PD, glucose 6-phosphate dehydrogenase; MDH, malate dehydrogenase; RP-A, rice protein extracted by alkaline treatment; RP-E, rice protein extracted by α-amylase.

After 2 weeks feeding, the activities of various enzymes involved in fatty acid synthesis (fatty acid synthase, glucose 6-phosphate dehydrogenase and malate dehydrogennase) were affected by dietary proteins (Figure 2). Compared with CAS, fatty acid synthase was markedly reduced by RP-A to a degree of -36.69% and by RP-E to -43.68% (*P *< 0.05) (Figure 2A). Similarly, the activities of glucose 6-phosphate dehydrogenase (Figure 2B) and malate dehydrogennase (Figure 2C) tended to be lower in RP-feeding groups than those fed CAS (*P *< 0.05).

**Figure 3:**
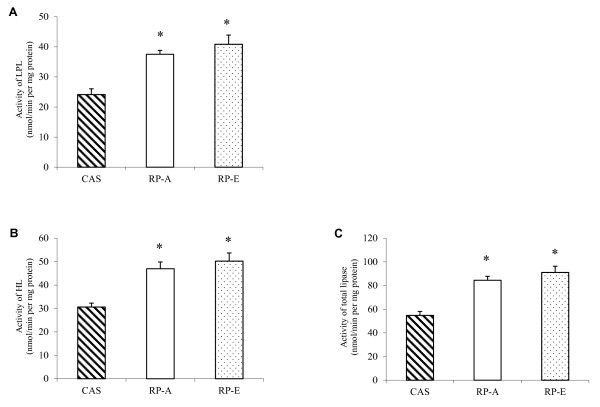
**Effects of dietary proteins on hepatic activities of lipolysis enzymes**. Hepatic activities of lipoprotein lipase (A), hepatic lipase (B) and total lipase (C) in 7-week-old male Wistar rats fed cholesterol-enriched diets for 2 weeks. Values are means ± SEM (n = 6). Bars for each value with * were significantly different from CAS (*P *< 0.05). CAS, casein; HL, hepatic lipase; LPL, lipoprotein lipase; RP-A, rice protein extracted by alkaline treatment; RP-E, rice protein extracted by α-amylase; TL, total lipase.

As illustrated in Figure 3, RP-A and RP-E exhibited the significant stimulation in activities of various lipases, which involve in the hydrolysis of triglyceride (*P *< 0.05). Compared with CAS, lipoprotein lipase was significantly stimulated by RP-feeding, accounting for 55.49% and 69.29% enhancements in RP-A and RP-E, respectively (Figure 3A). Similarly, the activity of hepatic lipase was 53.40% higher in growing rats fed RP-A, while RP-E caused a 1.64-fold increase, as compared with CAS (Figure 3B). As a result, the activity of total lipase in rats was significantly increased from 54.30% to 66.30% in RP-feeding (Figure 3C), as compared with CAS-feeding (*P *< 0.05).

### Fecal excretion

The 3-d fecal output was summarized in Table 6. Compared with CAS, RP-E produced marked fecal output by increasing 9.04% (*P *< 0.05), whereas fecal output in RP-A did not differ from CAS (*P *> 0.05). Unexpectedly, a significant difference of fecal output was found in RP-A and RP-E, implying a difference in the intestinal transport between these groups (*P *< 0.05).

**Table 6:** Fecal excretion of lipids and nitrogen in rats fed experimental diets^*a*^

	CAS	RP-A	RP-E
Fecal output (g dry wt/3 day)	3.87 ± 0.08^B^	3.91 ± 0.12^B^	4.22 ± 0.11^A^
Ffecal lipids			
total fat (mg/3 day)	222.39 ± 11.67^C^	296.26 ± 21.80^B^	360.42 ± 18.16^A^
triglyceride (μmol/3 day)	10.94 ± 0.30^C^	14.68 ± 0.67^B^	17.49 ± 0.89^A^
Fecal nitrogen (mg/3 day)	84.94 ± 1.43^C^	208.55 ± 5.24^B^	269.11 ± 5.74^A^
Fecal protein (g/3 day)	0.53 ± 0.01^C^	1.30 ± 0.03^B^	1.68 ± 0.04^A^
Intake of dietary protein (g/3 day)	9.76 ± 0.05	9.10 ± 0.25	9.12 ± 0.20
Apparent protein digestibility (%)	94.56 ± 0.11^A^	85.67 ± 0.29^B^	81.52 ± 0.59^C^

*^a^*Values are means ± SEM for six rats. Values with different letters are significant different (*P *< 0.05)

As shown in Table 6, fecal total fat excretion was significantly stimulated by RP-feeding (*P *< 0.05). Compared with CAS, fecal total fat excretion was 33.22% higher in growing rats fed RP-A, while RP-E caused a 1.62-fold increase. Among the lipids excreted, the concentration of triglyceride was also distinctly affected by RP-feeding, accounting for 34.19% and 59.87% enhancements in RP-A and RP-E, respectively, as compared with CAS (*P *< 0.05). Fecal excretions of total fat as well as triglyceride were significantly higher in RP-E than those in RP-A (*P *< 0.05), suggesting that the intestinal absorption of lipids appeared to be more effectively inhibited by RP-E than by RP-A.

In the RP groups, fecal nitrogen was significantly higher than that in the CAS group (*P *< 0.05), leading to the result that apparent protein digestibility was significantly lower in the RP groups as compared with CAS (*P *< 0.05) (Table 6). Results indicated that a marked difference of apparent protein digestibility was found in RP-E and RP-A, because the higher fecal nitrogen excretion was observed in RP-E than that in RP-A (*P *< 0.05), in accordance with the previous studies [15].

## Discussion

We examined the lipid-lowering potential of rice protein and especially the effects of rice protein on key enzymes involved in the lipolytic and lipogenic pathways of triglyceride metabolism. Our findings demonstrated that the triglyceride-lowering action of RP was associated with depressing fatty acid synthesis and stimulating triglyceride hydrolysis, suggesting that rice protein might possess a potential of anti-adiposity.

The most frequently suggested mechanism responsible for the lipid-lowering effect of plant protein is the interference with enterohepatic circulation, leading to an inhibition of hepatic secretion of lipids into circulation, which is closely associated with hepatic lipogenesis and lipoprotein production [17,26-28]. Yang *et al*. suggested that rice protein could depress the VLDL assembly and secretion to result in the decreased hepatic secretion of triglyceride and cholesterol into circulation, hence hypocholesterolemia and hypotriglyceridemia [19]. In the present study, results further confirmed and expanded this view. After 2 weeks feeding period, in addition to a significant reduction in hepatic accumulation of total lipids, cholesterol and triglyceride induced by RP-feeding, both RP-A and RP-E were found to be effective in depressing the activities of various enzymes involved in the regulation of fatty acids synthesis, showing the drastic reductions in the activities of fatty acid synthase, glucose 6-phosphate dehydrogenase and malate dehydrogenase. Furthermore, data obtained here indicated that there was a significant positive correlation between the activity of fatty acid synthase and the hepatic accumulation of triglyceride (r = 0.7939, *P *< 0.05), as well as the concentration of plasma VLDL-C (r = 0.7248, *P *< 0.05). As a results, a significant positive correlation between the activity of fatty acid synthase and the deposit fat (r = 0.8739, *P *< 0.05) was observed in this study. Thus, the decreased biosynthesis of fatty acids, in turn, will reduce the production of VLDL particles into circulation, hence leading to lower plasma triglyceride level and less deposit fat in the body. In accordance with the previous findings [29], results suggest that the lowering-effects on accumulation of lipids and deposit fat in rats were in part caused by the downregulation of activities of lipogenic enzymes, which also involved with the inhibition of hepatic assembly and secretion of VLDL.

Lipogenesis and lipolysis are two major processes for maintaining triglyceride homeostasis through regulatory triglyceride storage and triglyceride mobilization [16,17,30]. During lipolytic reaction, LPL is a rate-limiting enzyme in the provision of free fatty acid to muscles (utilization) and the adipose tissue (storage), thereby leading to weight loss or obesity [31]. Thus, the enzymolysis action of LPL may be an important factor participating in the accumulation of triglyceride.

Of interest was the finding in this study that the decreased concentrations of triglyceride and free fatty acid in the plasma were found in RP groups, despite the observed increment of the activities of LPL and HL induced by RP-A and RP-E. Also, compared with CAS, we did not observe the plasma VLDL-elevating effect of RP-feeding. Thus, the question arises why the stronger stimulation of triglyceride hydrolysis and the more concentration of free fatty acids, which were induced by higher activity of LPL and HL, could not result in the excess accumulation of triglyceride and free fatty acid in plasma. The precise mechanism by which RP affects triglyceride metabolism is not fully understood, but the mechanism responsible for the effect of LPL and HL on the removal of VLDL from circulation by hepatic uptake should be taken into account [16-18]. Besides its hydrolytic activity, LPL and HL might interact with lipoproteins to anchor them the vessel wall and facilitate lipoprotein particle uptake by the liver. Particularly, LPL and HL are also important in HDL metabolism contributing to the transfer of surface lipid to small HDL after lipolysis [17,18]. In addition, it is noteworthy that the decreased fatty acid synthesis in this study was unable to completely compensate for the marked depletion of hepatic triglyceride for the hydrolysis and the secretion into circulation via VLDL. To support this view, Yang *et al*. provided direct evidence by the perfused rat liver under the same experimental condition that hepatic triglyceride secretion into circulation as well as hepatic triglyceride secretion into VLDL were effectively depressed by RP-feeding, showing a decrease of the availability of hepatic triglyceride for VLDL secretion into circulation [19]. Clearly, the finding observed in this study suggests that, in addition to the proposed mechanism of inhibition of fatty acid synthesis and the stimulation of triglyceride oxidation, the uptake lipoprotein may participate in the triglyceride-lowering action of RP, where HDL is the major plasma lipoprotein.

Also, compared with CAS, excess accumulation of deposit fat was not observed in RP groups, indicating less free fatty acid was used for storage in rats fed RP-A and RP-E, despite the stimulation of hydrolysis of triglyceride. To explain this phenomenon, some studies suggest that weight loss selectively increase adipose tissue LPL activity as an attempt to maintain lipid stores, resulting in a diminish in fat mass [18,31]. Thus, in light with this view, results indicated that lower deposit fat and weight loss in rats under the present experimental condition was closely associated with higher activity of LPL. Furthermore, a significant negative correlation between the activity of LPL and the weight of abdominal fat (r = -0.8761, *P *< 0.05), as well as the concentration of plasma triglyceride (r = -0.8410, *P *< 0.05) was found in rats fed cholesterol-enriched diets. Taken together, results obtained in this study clearly suggest that rice protein may possess a vital function in improving lipids level and adiposity.

The causes of the lipid-lowering action of rice protein, especially resistant to adiposity, appear to be multi-factorial. It has been demonstrated that the biological utilization of a protein is primarily dependent on its digestibility by gastric, pancreatic, and intestinal peptidases [32,33], providing the insight that the digestibility of rice protein may be a major factor to influence lipid metabolism. To support this view, in the present study, the lower intestinal absorption of fat and triglyceride caused by higher indigestible proportion in the intestine was found in RP-feeding, showing a significant negative correlation between the apparent protein digestibility and the fecal excretion of total fat (r = -0.8824, *P *< 0.05), as well as the fecal triglyceride excretion (r = -0.9165, *P *< 0.05). As a results, a significant positive correlation between the apparent protein digestibility and the deposit fat (r = 0.8567, *P *< 0.05) as well as the plasma TG concentration (r = 0.8627, *P *< 0.05) was observed in this study. In addition, the inhibition of activities of ALT and AST in plasma was also investigated RP-A and RP-E, indicating that RP-feeding could efficaciously improve liver histology and result in less fatty infiltration in hepatocytes as compared to CAS. On the other hand, the amino acid compositions can be influenced by the extraction method. The contents of the first limiting amino acid-Lys and the second limiting amino acid-Thr in RP-A and RP-E were lower than those in CAS. As a result, the higher ratio of Arg/Lys, which can regulate the digestibility of rice protein [12], was found in RP-A (2.56) and in RP-E (3.15) than that in CAS (0.44). Thus, the body-fat lowering response to RP may be in part attributable to their lower digestibility. Clearly, the precise mechanism remains to be clarified in further studies.

Here, it must be noted that the association of changes in the metabolism of branched chain amino acids (BCAAs) with alteration of lipids level was observed under the present experimental condition. A significant positive correlation between the plasma level of BCAAs and the weight of body fat (r = 0.7316, *P *< 0.05), as well as the gain in body weight (r = 0.7270, *P *< 0.05) was investigated in this study. The observed changes induced by RP-E tended to be the lowest level of plasma BCAAs among the experimental groups (RP-E: 466.5 nmol/mL; RP-A: 482.5 nmol/mL; CAS: 575.2 nmol/mL), showing the similar tendency with plasma triglyceride (r = 0.7958, *P *< 0.05). Indeed, an improved understanding of the mechanism underlying obesity-related rises in plasma BCAAs is important. It has been demonstrated that obesity-related elevations in plasma leucine are associated with alterations in enzymes involved in BCAA metabolism [34]. In addition, raises in plasma BCAAs due to a block in mitochondrial branched chain amino acid transaminase (BCATm) have been also associated with improvement in glucose metabolism [34]. Thus, an idea, which is consistent with previous studies, is that a reduction in plasma concentrations of BCAAs induced by RP-feeding might represent a possible mechanism for improving body weight control and resistant adiposity in rats fed cholesterol-enriched diets. Clearly, additional studies are required to confirm this view.

## Conclusion

In conclusion, the present study demonstrates that rice protein possesses a vital function in the modification of triglyceride metabolism, leading to an improvement of body weight and adiposity. The triglyceride-lowering action as well as the potential of anti-adiposity induced by rice protein is attributed to the upregulation of lipolysis and the downregulation of lipogenesis. Results suggest that the inhibition of lipid absorption, which is closely associated with the lower digestibility, may be the main modulator responsible for the triglyceride-lowering action of rice protein. The precise mechanisms involved in the anti-adiposity responses to rice protein await more detailed investigation in further study.

## Abbreviations

ALT: Alanine transaminase; AST: Aspartate transaminase; BCAA: Branched chain amino acid; CAS: Casein; EAA: Essential amino acid; FAS: Fatty acid synthase; FFA: Free fatty acid; G6PD: Glucose 6-phosphate dehydrogenase; HDL-C: High-density lipoprotein cholesterol; HL: Hepatic lipase; LDL-C: Low-density lipoprotein cholesterol; LPL: Lipoprotein lipase; MDH: Malate dehydrogenase; RP: Rice protein; RP-A: Rice protein extracted by alkaline; RP-E: Rice protein extracted by α-amylase; TC: Total cholesterol; TG: Triglyceride; TL: Total lipase; VLDL: Very-low-density lipoprotein.

## Competing interests

The authors declare that they have no competing interests.

## Authors' contributions

LY designed the study, conducted the study and wrote the manuscript. JHC supervised the study and conducted statistical analysis. JL and QW conducted statistical analysis and revised the manuscript. TX carried out the study and analyzed lipids in plasma and liver. HZ and QHL analyzed the activities of hepatic enzymes. HKY conducted the experiments and analyzed the fecal fat. LY had primary responsibility for final content. All authors read and approved the final manuscript.
